# Choice of refractive surgery types for myopia assisted by machine learning based on doctors’ surgical selection data

**DOI:** 10.1186/s12911-024-02451-0

**Published:** 2024-02-08

**Authors:** Jiajing Li, Yuanyuan Dai, Zhicheng Mu, Zhonghai Wang, Juan Meng, Tao Meng, Jimin Wang

**Affiliations:** 1 School of Artificial Intelligence, China University of Mining and Technology (Beijing), Beijing, China; 2 Wangganzhicha Information Technology Inc., Nanjing, Jiangsu Province China; 3 Department of Ophthalmology, Peking Union Medical College Hospital, Chinese Academy of Medical Sciences, Beijing, China; 4 Key Laboratory of Ocular Fundus Diseases, Chinese Academy of Medical Sciences & Peking Union Medical College, Beijing, China; 5 Community Health Service Center of Douhudi Town, Gongan County, Jingzhou, Hubei Province China; 6 Department of Information Management, Peking University, Beijing, China

**Keywords:** Data mining, Machine learning, Refractive surgery, Random forest, SMOTE

## Abstract

In recent years, corneal refractive surgery has been widely used in clinics as an effective means to restore vision and improve the quality of life. When choosing myopia-refractive surgery, it is necessary to comprehensively consider the differences in equipment and technology as well as the specificity of individual patients, which heavily depend on the experience of ophthalmologists. In our study, we took advantage of machine learning to learn about the experience of ophthalmologists in decision-making and assist them in the choice of corneal refractive surgery in a new case. Our study was based on the clinical data of 7,081 patients who underwent corneal refractive surgery between 2000 and 2017 at the Department of Ophthalmology, Peking Union Medical College Hospital, Chinese Academy of Medical Sciences. Due to the long data period, there were data losses and errors in this dataset. First, we cleaned the data and deleted the samples of key data loss. Then, patients were divided into three groups according to the type of surgery, after which we used SMOTE technology to eliminate imbalance between groups. Six statistical machine learning models, including NBM, RF, AdaBoost, XGBoost, BP neural network, and DBN were selected, and a ten-fold cross-validation and grid search were used to determine the optimal hyperparameters for better performance. When tested on the dataset, the multi-class RF model showed the best performance, with agreement with ophthalmologist decisions as high as 0.8775 and Macro F1 as high as 0.8019. Furthermore, the results of the feature importance analysis based on the SHAP technique were consistent with an ophthalmologist’s practical experience. Our research will assist ophthalmologists in choosing appropriate types of refractive surgery and will have beneficial clinical effects.

## Introduction

Myopia is characterised by high prevalence, low age, and rapid progression, making it a global public health problem. In recent years, corneal refractive surgery has been widely used in clinics as an effective means to restore vision and improve the quality of life. Many clinical and basic experimental studies confirmed its safety [1], effectiveness, stability, and predictability [2].

Currently, frame lenses, contact lenses, corneal refractive surgery, and intraocular lens implant surgery are the main methods to correct refractive errors. The principle is to correct the light entering the human eye by diverging it and focusing it on the retina. For teenagers whose eyeballs are not fully developed and whose refractive power is unstable, or for patients who cannot undergo surgery, refractive correction is mainly done by wearing glasses, contact lenses, phakic IOL implantation [3], or vision rehabilitation training. For adults who meet the criteria for laser vision correction surgery, correction can be achieved through corneal refractive surgery or intraocular lens surgery. At present, the corneal refractive surgeries clinically used in my country include laser in situ keratectomy (LASIK), laser photo keratectomy (PRK), and SMall Incision Lenticule Extraction (SMILE). The diversification of surgical methods has brought new surgical techniques, outcomes, and options to patients and operators, as well as improved surgical outcomes, safer surgical procedures, and fewer surgical complications.

Since the first reported case of corneal dilatation after refractive surgery [4], the safety of refractive surgery has attracted the attention of physicians. Because the operation changes the normal anatomical structure of the cornea, there may be complications such as epithelial or corneal stroma opaque bubbles, difficulty in opening the corneal flap, postoperative transient photosensitivity syndrome, and incomplete lens removal during femtosecond laser surgery [5]. Corneal flap abnormality, displacement, folds, postoperative epithelial implantation, and other special circumstances may occur during and after surgery [6]. Complications of postoperative myopia itself, such as fundus hemorrhage, retinal degeneration, retinal detachment, and glaucoma, may still occur [7]; Postoperative complications may also occur, such as refractory dry eye, decreased scotopic vision, phantom vision, ghosting, glare [8]. It is also possible that reoperation is required because of deviations in the surgical type or parameters. Therefore, ophthalmologists must be cautious in screening patients reasonably, scientifically, and rationally before surgery and choose the most suitable surgical method for patients to achieve ideal surgical results and reduce surgical risks.

Some studies have used artificial intelligence to assist decision-making in myopia corneal refractive surgery [9–13]. Among them, Balidis M et al. [12] utilized neural networks to predict the need for retreatment after refractive surgery for myopia, achieving statistically significant predictions with good sensitivity (0.8756) and specificity (0.9286). Melles RB et al. [13] proved that artificial intelligence is more helpful in the calculation of the refractive IOL degree and the quantification of the position in intraocular refractive surgery. The datasets of these researches typically have a short time span and only contain a limited set of surgical types. In our study, we used machine learning to learn about the experience of ophthalmologists in decision-making and assist them in the choice of corneal refractive surgery in a new case. Our research was based on the data of 7,081 patients who underwent surgery at the Department of Ophthalmology, Peking Union Medical College Hospital, Chinese Academy of Medical Sciences between 2000 and 2017. The patients were divided into three groups according to the type of surgery performed: LASIK, PRK, and SMILE. Our research explored how to perform data preprocessing, feature selection, and machine learning model training to achieve the best prediction performance. The contributions of this study are as follows:

1. Aiming at the preprocessing of ophthalmology data, we provided data cleaning, feature selection, as well as a small number of oversampling techniques (SMOTE) to deal with the data imbalance problem in the experimental data.
2. We selected and trained six statistical machine learning models, including the Naive Bayesian Model (NBM), Random Forest (RF), Adaptive Boosting (AdaBoost), eXtreme Gradient Boosting (XGBoost), Back Propagation Neural Network (BP Neural Network), and Deep Belief Network (DBN), and used ten-fold cross-validation and grid search to find the optimal hyperparameters to improve the accuracy of the classification model. When tested on the dataset, the multi-class RF model showed the best performance, with agreement with ophthalmologist decisions as high as 0.8775 and Macro F1 as high as 0.8019. The model results showed that the model had good clinical application value.
3. To verify the rationality of feature selection, we used SHAP [14] to quantify the importance of features. The results were highly consistent with the practical experience of ophthalmologists. Ultimately, the developed model is able to provide confidence to doctors and patients by recommending surgery based on data when deciding on a surgical method.

## Related work

Since 1970, artificial intelligence (AI) research has made certain breakthroughs in the medical field, and a series of clinical decision support models have been used in the diagnosis of ophthalmic diseases and the choice of treatment options, which can involve retinal images, optical coherence tomography (OCT), slit lamp images and other data that can be automatically analysed and applied to glaucoma [15, 16], age-related macular degeneration [17], cataract [18], Keratoconus [19], dry eye disease [20] screening for eye diseases, diabetic retinopathy [21], diabetic peripheral neuropathy [22] and auxiliary diagnosis of ocular manifestations such as systemic diseases [23]. In refractive surgery, the advantages of AI mainly focus on keratoconus screening, selection of refractive surgery options, design of surgical parameters (nomogram), postoperative efficacy prediction, and implantable intraocular lens implantation in phakic eyes. Collamer lens (ICL) postoperative arch height prediction and other aspects have also been explored [24–28].

In the clinical practice of refractive surgery, Yoo TK et al. [29] established a machine learning architecture that combines a large number of different instrument data from patients and the clinical decisions of experienced experts to analyse the possible impact on surgical results, to identify surgical candidates and avoid surgical complications that may happen. In addition, Achiron A et al. [30] extracted 38 clinical parameters from the case data of 17,592 patients who underwent LASIK or PRK surgery in an ophthalmology department for 12 consecutive years and used the statistical classifier algorithm to train and test the machine learning classifier. The study found that surgical effectiveness decreased with age, central corneal thickness, average corneal curvature, and preoperative CDVA, but increased with pupil size. Cui T et al. [31] used 1,146 eye sample data and the MLPNN algorithm to construct a Nomogram prediction model for SMILE surgery based on machine learning algorithms and compared the ML model with clinical experts. There were no significant differences; however, the ML group was superior to the clinical expert group in terms of efficacy and predictability. The postoperative spherical equivalent dioptres of the ML group and the clinical expert group are -0.09 ± 0.024 and -0.23 ± 0.021, respectively.

Yoo TK et al. [32] developed an interpretable multi-category XGBoost model for the expert-level choice of refractive surgery, classifying patients into four types: laser epithelial keratopathy, laser in situ keratopathy, small-incision lens extraction, and contraindications. The analysis included 18,480 subjects and 142 variables, and the model achieved accuracies of 81.0% and 78.9% when tested on the internal and external validation datasets, respectively. Our study verified the results of the paper conducted by Yoo TK et al. [32], but this study has the following differences and improvements: (1) Aiming at the preprocessing of ophthalmology data, we provided data cleaning, feature selection, as well as a small number of oversampling techniques (SMOTE) to deal with the data imbalance problem in the experimental data. (2) For the selection of machine learning models, we investigated 15 mainstream machine learning models in the early stage. We finally selected and trained six statistical machine learning models, including the NBM, RF, AdaBoost, XGBoost, BP Neural Network, and DBN, and used ten-fold cross-validation and grid search to find the optimal hyperparameters to improve the accuracy of the classification model. (3) Our data set Clinical data was collected from patients who underwent corneal refractive surgery at the Peking Union Medical College Hospital of the Chinese Academy of Medical Sciences from January 2000 to October 2017. The time span is very long, more than 18 years. Combined with the previous examination methods, which were very limited, the well-preserved data and conducting research is of great cross-epochal significance. (4)In our experiments, it was discovered and verified that the feature of “sphere-column conversion” had a certain degree of influence on the predicted results of the surgical method, which has new clinical application value.

The theory and technology of corneal refractive surgery are becoming increasingly perfect; however, there are still problems such as preoperative screening difficulties and postoperative complications in clinical practice, and the safety and accuracy of surgery still need to be further improved. In addition, due to the lack of data transparency, it is impossible to critically evaluate the quality of the model [33]. There are various sources of clinical data for building AI models, and there is no unified standard, which also limits the development of AI [34]. When the diagnosis and treatment opinions of the disease are inconsistent, the accuracy of AI prediction is affected. Due to the black-box nature of the algorithm, the previous AI model cannot make a reasonable explanation for the decision-making like human experts [35]. However, this study uses visualization technology to explain the results of the multi-classification RF algorithm. The developed model can recommend surgeries based on data when deciding on surgical methods. The consistency with the clinical decision-making of ophthalmologists is as high as 87.75%, providing confidence to doctors and patients.

## Data preparation

The dataset for this study was obtained from the clinical data of patients who underwent corneal refractive surgery at the Peking Union Medical College Hospital, Chinese Academy of Medical Sciences, from January 2000 to October 2017, and all data have been desensitized. This study was approved by the Ethics Review Committee of Peking Union Medical College Hospital, Chinese Academy of Medical Sciences. All methods were carried out in accordance with relevant guidelines and regulations in the Declaration of Helsinki. All participants signed an informed consent. At this ophthalmic centre, corneal refractive surgery is considered the main method of refractive error correction.

Previous studies have shown that Reinstein DZ et al. [36] found that the postoperative TTS is considerably higher after SMILE than both PRK and LASIK, because the strongest anterior lamellae remains intact. Consequently, SMILE should be able to correct higher levels of myopia. Xin Y et al. [37] found that the corneal biomechanical response to the three surgical procedures varied significantly. With similar corneal thickness loss, the reductions in overall corneal stiffness were the highest in FS-LASIK and the lowest in tPRK. The effective remaining corneal volume of different corneal refractive surgeries (such as LASIK, PRK, SMILE) is different and their biomechanical properties are different. Based on previous research and combined with the surgical experience of senior experts from Union Medical College, we added the criteria for whether laser vision correction surgery is feasible into the model training by hand-crafted rules. First, patients who could not undergo refractive surgery were excluded. The exclusion criteria included corneal diseases, such as keratoconus and corneal dilation disease; autoimmune diseases; systemic model diseases or partial metabolic diseases, such as severe hyperthyroidism or hyperthyroid exophthalmos; and active eye lesions, such as intraocular and corneal infections; poor fundus function, or severe cataract, glaucoma and other eye diseases; diabetes mellitus; cicatricial constitution; data loss >10%; refusing surgery during or after the examination.

All patients who pass the above screening are required to undergo strict eye examination before surgery. The preoperative examination and main instruments include: automatic computer refraction (Topcon, RM-800, Japan); Subjective refractor (Topcon, CV-5000, Japan) measured distance uncorrected visual acuity and distance corrected visual acuity; non-contact intraocular pressure measurement (Canon, TX-20, Japan); A fully automatic non-contact tonometer (TXF, Canon Company, Japan) was used to measure intraocular pressure, and a slit lamp microscope (BQ900, HAAG-STREIT Company, Switzerland) was used for examination; Corneal topography (Oculus, Pentacam, Germany) examination; ocular wavefront aberrometer (Visx4, United States), all the above examinations were completed by the same experienced optometrist. Doctors conduct a preliminary screening of surgical methods according to simple criteria. General criteria for considering surgery include the following parameters: age 18 years or older; preoperative diopter: spherical diopter (SD) $\le$ -9.00 diopters (D), astigmatism (CD) $\le$ -3.00D, and the diopter is in a stable state within 2 years; intraocular pressure (IOP): 10-21mmHg; spherical equivalent < +6.0D; central corneal thickness (CCT) measured by pachymetry is required, >500$\mu$m for LASIK, >480$\mu$m for SMILE and >460$\mu$m for PRK; and surgery posterior residual corneal thickness >280$\mu$m.

Subsequently, this study used the following expert knowledge and added them to the training of the model in the form of hand-crafted rule: for patients with thin corneas and high refractive power, PRK is preferred [38]; SMILE is not recommended for patients with high astigmatism [39]; SMILE is not recommended for patients with irregular corneas; Myopic patients with a history of high intraocular pressure or glaucoma should avoid superficial surgery; patients with severe dry eye should avoid LASIK surgery. The type of surgery is determined based on the surgical experience and actual situation of senior experts from the Chinese Academy of Medical Sciences and Peking Union Medical College. And using machine learning methods to learn and combine various judgment factors to ultimately determine the type of refractive corneal surgery that is most suitable for each eye. It should be noted that those who could not be corrected all at once because of high myopia and insufficient corneal thickness needed to maintain a certain degree of vision, and were required to wear glasses after surgery to achieve the best vision.

At least we got the dataset with the clinical data of 7,081 patients. In the dataset, there are three situations: the same operation is performed on both eyes simultaneously, different operations are performed on both eyes simultaneously, and the operation is performed on one eye. In the end, we obtained a total of 13,723 pieces of data in the data set, including 6,872 left-eye data and 6,851 right-eye data. The model predicted the left eye and right eye respectively.

As shown in Table 1, three types of laser corneal refractive surgeries are currently mainstream for correcting refractive errors, all of which have good predictability and safety. Among them, LASIK, PRK, and SMILE can perform individualised ablation guided by wavefront aberration, Q value, and corneal topography, which are beneficial for improving the visual quality of patients [40]. The most commonly used surgical methods are LASIK and SMILE. This is because LASIK technology is very mature, has been practiced for many years, has a very good reputation, and doctors have a wealth of experience. Furthermore, SMILE represents the latest development in the field of corneal refractive surgery and is the most popular surgery in recent years. The clinical implementation time is relatively short; however, it has completely realised the leap of minimally invasive and flapless refractive surgery [41], avoiding potential risks such as corneal flap folds, displacement and loss, and bringing corneal refractive surgery into the era of femtoseconds [42].

**Table 1:** Technical characteristics and sample size of corneal refractive surgery for cutting

Surgical Method	Features	Number of Samples
LASIK	It uses a microkeratome to make a pedicled lamellar corneal flap with a diameter of 8^∼^10mm and a thickness of 130^∼^160um. An excimer laser is used to cut a concave surface with a certain diopter on the stromal bed, and then the corneal flap is reset. By changing the corneal front the curvature of the surface achieves the purpose of correcting myopia [43].	9096
PRK	There is no need for a mechanical blade or ethanol to remove the corneal epithelium. The laser directly cuts the epithelium and stromal layer on the corneal surface, and no flap is required during the operation [44].	1991
SMILE	First, a femtosecond laser is used to create a lens in the corneal stroma, then a tiny incision is made to avoid the creation of a corneal flap, and finally, the corneal stromal lens tissue is separated in a small incision of 2-5mm [45].	2636

Table 2 shows twenty attributes for patients which were extracted from demographic characteristics, physical examination report, corneal biomechanical properties, ophthalmological measurements and interview questionnaire, etc, namely fBestEye (Vision with glasses), fNudeEye (Vision without glasses), Central Corneal Thickness, Intraocular Pressure, DS Spherical Power, DC Cylinder Power, fDS3 (Cylinder Axis), Re-examination Optometry, fDC2 (Recheck Cylinder), fDC3 (Recheck Axis), Pupil, Dilated Pupil, Sphere-column Conversion, Corneal Curvature K1, Axis K1, Corneal Curvature K1, Axis K2, fSRI (Surface Regularity Index), fSAI (Corneal Asymmetry Index), and fCSI (Corneal Spherical Aberration). Among them, Sphere-column Conversion = spherical equivalent refraction * astigmatism / 2. These attributes help us determine which type of refractive surgery is selected as the best one for an eye. Due to the fact that the data in this paper is divided into three categories, the comparison was conducted using the Kruskal-Wallis test, with P<0.05 indicating a statistically significant difference.

**Table 2:** Features statistics of corneal refractive surgery prediction models

Feature Name (unit)	LASIK	PRK	SMILE	*P* Value
fBestEye/(logMAR) (Vision with glasses)	0.03(±1.03)	0.03(±1.03)	0.00(±1.00)	<0.01
fNudeEye/(logMAR) (Vision without glasses)	0.85(±0.55)	0.85(±0.55)	0.77(±0.47)	<0.01
Central Corneal Thickness/($\mu$m)	539.31(±151.06)	537.84(±107.16)	539.04(±108.96)	<0.01
Intraocular Pressure/(mmHg)	15.79(±15.21)	15.75(±9.25)	15.65(±11.45)	<0.01
DS Spherical Power/(D)	-5.28(±14.28)	-4.93(±12.43)	-5.26(±7.99)	<0.01
DC Cylinder Power/(D)	-1.29(±7.29)	-1.22(±7.22)	-1.24(±7.24)	<0.01
fDS3(Cylinder Axis)/(D)	117.76(±62.24)	114.71(±65.29)	115.23(±64.77)	<0.01
Re-examination Optometry/(D)	-5.49(±15.49)	-5.03(±15.03)	-5.51(±14.51)	<0.01
fDC2 (Recheck Cylinder)/(D)	-1.22(±7.22)	-1.23(±7.23)	-1.23(±7.23)	<0.01
fDC3 (Recheck Axis)/(D)	116.98(±62.02)	115.35(±64.65)	115.88(±64.12)	<0.01
Pupil/(mm)	2.70(±1.80)	2.69(±1.31)	2.71(±1.30)	<0.01
Dilated Pupil/(mm)	5.26(±1.26)	5.34(±1.34)	5.29(±1.15)	<0.01
Sphere-column Conversion/(D)	-6.18(±19.43)	-5.29(±18.29)	-5.99(±18.99)	<0.01
Corneal Curvature K1/(D)	44.28(±10.14)	44.26(±12.11)	44.31(±10.12)	<0.01
Axis K1/(D)	90.83(±80.83)	90.57(±80.57)	90.62(±80.04)	<0.01
Corneal Curvature K2/(D)	43.05(±11.01)	43.09(±11.05)	43.07(±11.08)	<0.01
Axis K2/(D)	98.35(±81.65)	99.92(±80.08)	98.35(±81.65)	<0.01
fSRI (Surface Regularity Index)/(D)	0.20(±0.83)	0.20(±0.95)	0.20(±0.85)	<0.01
fSAI (Corneal Asymmetry Index)/(D)	0.32(±0.91)	0.32(±1.19)	0.34(±0.93)	<0.01
fCSI (Corneal Spherical Aberration)/($\mu$m)	1.34(±4.04)	1.26(±3.86)	1.33(±4.07)	<0.01

## Experiments and discussion

In this section, we present our experimental methods and conclusions, including the data preprocessing, predictive model selection and training, and feature importance analyses.

### Data preprocessing

Owing to the features of irregularity, high dimensionality, redundancy, and data loss in our dataset, a series of preprocessing steps were performed on the original data before data mining, including 1) data cleaning to identify outliers and duplicates and 2) deleting cases lacking key values.

The experimental data used in our study had multiple features, each with different dimensions and dimension units. Differences in the magnitude of the feature data can affect the performance of the model. For example, features at different scales may lead the model to pay more attention to features with larger values, thus ignoring other important features and leading to a decrease in its predictive performance. Therefore, we further standardized and normalized the data to increase the solution speed of the gradient descent and eliminate the influence of the magnitude and dimension, thereby improving the convergence speed and accuracy of the prediction model. Standardization refers to Z-core normalization, which enables the values of all features to be converted into a normal distribution with a mean of zero and a standard deviation of one. Normalization refers to min-max scaling, which converts each feature value into a [0,1] interval. For each feature, the minimum and maximum values were converted to 0 and 1, respectively. Deviation standardization can transform data into different proportions, eliminate the dominance of special features, and does not require assumptions regarding the distribution of data. However, normalization cannot handle outliers. In contrast, standardization can better handle outliers and accelerate the convergence of algorithms, such as gradient descent. This study selected the optimal data preprocessing method by comparing the classification effects of standardization and normalization.

Our experimental data showed an imbalance in the number of cases for the three surgical methods. Among them, LASIK had the largest number of cases (9,096), and PRK had the smallest number of cases (1,991). In this study, a synthetic minority oversampling technique (SMOTE) [14] was adopted to overcome the imbalance problem. The SMOTE method is an oversampling method that randomly generates new instances of minority classes to balance the number of classes and is the most popular and effective method for balancing the dataset during training. When generating binary variables (gender or yes/no questionnaire) using SMOTE, a rounding function was applied after the SMOTE process to restore the binary variable attributes. Subsequently, a fully balanced dataset was generated using the SMOTE technique, such that the surgical modalities in the experimental data had the same number of instances.

### Model training

In our study, the choice of surgical type was regarded as a classification problem, and the aim was to develop an optimal classification model based on the dataset. Six statistical machine learning methods, namely NBM, RF, AdaBoost, XGBoost, BP neural network, and DBN were selected. The dataset was randomly divided into training (80%, n=10978) and testing (20%, n=2745). Ten-fold cross-validation and grid search are used to find the optimal hyperparameters, and then SMOTE is used for oversampling in each cross-validation cycle to ensure the accuracy of the validation results. In the experiment, feature selection refers to selecting the first 12 features with feature importance greater than 0.4 for model training. We conducted model training by conducting two sets of comparative experiments on the left and right eyes respectively through feature selection and max_depth parameter selection.

The six machine learning models are based on different design concepts and technical principles. The settings of the public parameters in the model are as follows, the random seed is set to 1, the number of iterations max_iter is set to 1000, and the activation function of the hidden layer is set to relu.

1. The NBM [46] model, which classifies by calculating probability, is suitable for multi-classification tasks and incremental training. For large-scale data, the computational complexity is low, and the algorithm principle is relatively simple and easy to understand.
2. The RF [47] model combines the classification results of several weak classifiers to form a strong classifier. It can evaluate the importance of each feature in the classification problem, can effectively run the input samples of high-dimensional features, does not require dimensionality reduction, has excellent accuracy, and can also obtain good results for missing value problems.
3. The AdaBoost [48] model is an iterative algorithm. Its core idea is to train different classifiers (weak classifiers) for the same training set, and then combine these weak classifiers to form a stronger final classifier (strong classifier).
4. XGBoost [49] is an improvement to the gradient boosting algorithm. Newton’s method is used to solve the extreme value of the loss function, and the loss function Taylor is expanded to the second order. In addition, a regularization term is added to the loss function.
5. The BP neural network [50] model has arbitrarily complex pattern classification capabilities and excellent multi-dimensional function mapping capabilities, and solves XOR and some other problems that simple perceptrons cannot solve.
6. The DBN [51] model is a hybrid model composed of a restricted Boltzmann machine (RBM) and a sigmoid belief network (SBN). Compared with the neural network of the traditional discriminant model, it establishes a joint distribution between observation data and labels, in which Both P (Observation | Label) and P (Label | Observation) are evaluated.

### Experiment results

We use the currently recognized general indicator accuracy [52] and Macro-F1 as indicators for evaluating model performance. Accuracy is defined as the percentage of samples correctly classified by the prediction model in all samples, reflecting the ability of the prediction model to identify if various samples and the formula is shown in (1), where TP(True Positives) represents the number of correct predictions for positive samples, TN(True Negatives) represents the number of correct predictions for negative samples, and N represents the total number of samples.

1 $$\begin{aligned} \text {Accuracy} = \frac{\text {TP}+\text {TN}}{N} \end{aligned}$$

The Macro-F1 reflects the model’s performance in multi-category classification tasks, especially when dealing with imbalanced category distributions. It measures the model’s ability to recognize each category and calculates the weighted average of its precision and recall across all categories, as shown in formulas (2) and (3):

2 $$\begin{aligned} \text {Precision}_{\text {macro}}=\frac{\sum _{\text {i}=1}^{\text {n}} \text {Precision}_{\text {i}}}{\text {n}} \end{aligned}$$

3 $$\begin{aligned} \text {Recall}_{\text {macro}}=\frac{\sum _{\text {i}=1}^{\text {n}} \text {Recall}_{\text {i}}}{\text {n}} \end{aligned}$$

The Macro-F1 is calculated using formula (4):

4 $$\begin{aligned} \text {F} 1_{\text {macro}}=2 \cdot \frac{\text {Precision}_{\text {macro}} \cdot \text {Recall}_{\text {macro}}}{\text {Precision}_{\text {macro}}+\text {Recall}_{\text {macro}}} \end{aligned}$$

### Data preprocessing and experimental results

As mentioned earlier, the data processing stage includes data cleaning, SMOTE technology, and feature selection. Among them, the SMOTE [53] method is used to deal with the data imbalance problem by randomly generating new instances of a minority class. Feature selection is performed by selecting the top 12 features with importance greater than 1.4%. In this section, we conduct multiple comparative experiments to verify the effectiveness of the hyperparameter max_depth and feature processing on classification performance. Tables 3 and 4 show the performance of the machine learning model predictions for the left and right eyes, respectively, ACC and Macro-F1.

**Table 3:** Accuracy and Macro_F1 of machine learning models on left eye data with different hyperparameters max_depth and with/without feature selection

	Model	Max_depth					
		9		10		11	
		ACC	Macro_F1	ACC	Macro_F1	ACC	Macro_F1
Feature selection (Select the top 12 features with importance greater than 1.4%.)	NBM	0.7566	0.5696	0.7566	0.5696	0.7566	0.5696
	DBN	0.8276	0.5252	0.8276	0.5252	0.8276	0.5252
	RF	**0.8676**	**0.7635**	**0.8775**	**0.8019**	**0.8725**	**0.7778**
	AdaBoost	0.8374	0.6970	0.8424	0.6871	0.8079	0.6514
	XGBoost	0.8676	0.7460	0.8677	0.7484	0.8725	0.7603
	BP Neural Network	0.8578	0.7462	0.8578	0.7462	0.8578	0.7462
No feature selection was performed (There are 20 features in total.)	NBM	0.6946	0.5643	0.6946	0.5643	0.6946	0.5643
	DBN	0.8054	0.5165	0.8054	0.5165	0.8054	0.5165
	RF	0.8172	0.7148	0.8173	0.6998	0.8226	0.7117
	AdaBoost	0.7784	0.6348	0.7622	0.6284	0.7514	0.6191
	XGBoost	0.8118	0.6872	0.8172	0.6998	0.8226	0.7117
	BP Neural Network	0.7957	0.6271	0.7957	0.6271	0.7957	0.6271

**Table 4:** Accuracy and Macro_F1 of machine learning models on right eye data with different hyperparameters max_depth and with/without feature selection

	Model	Max_depth					
		9		10		11	
		ACC	Macro_F1	ACC	Macro_F1	ACC	Macro_F1
Feature selection (Select the top 12 features with importance greater than 1.4%.)	NBM	0.7435	0.4633	0.7435	0.4633	0.7435	0.4633
	DBN	0.7487	0.4824	0.7487	0.4824	0.7487	0.4824
	RF	**0.8125**	**0.7694**	**0.8229**	**0.8080**	**0.8073**	**0.7527**
	AdaBoost	0.7644	0.6475	0.7382	0.6047	0.7330	0.5931
	XGBoost	0.8020	0.7373	0.7917	0.7102	0.7917	0.7084
	BP Neural Network	0.7708	0.6621	0.7708	0.6621	0.7708	0.6621
No feature selection was performed (There are 20 features in total.)	NBM	0.7487	0.4657	0.7487	0.4657	0.7487	0.4657
	DBN	0.7435	0.4262	0.7435	0.4262	0.7435	0.4262
	RF	0.8177	0.7998	0.8021	0.7427	0.8073	0.7527
	AdaBoost	0.7435	0.6411	0.7592	0.6428	0.7487	0.6337
	XGBoost	0.7969	0.7182	0.8073	0.7426	0.7969	0.7203
	BP Neural Network	0.7760	0.6818	0.7760	0.6818	0.7760	0.6818

From Tables 3 and 4, it can be seen that:

1. According to the results of the two sets of comparative experiments, the best method is to select the first 12 features with feature importance greater than 1.4% and the RF model trained when max_depth=10. The accuracies of the left eye and right eye are 0.8775 and 0.8229 respectively, and the Macro-F1 is 0.8019 and 0.8080 respectively. The model’s performance demonstrates its significant clinical usability. The prediction performance of the DBN model without feature selection is relatively the lowest. The accuracy of the left eye and right eye are 0.6946 and 0.7487 respectively, and the Macro-F1 is 0.5643 and 0.4657 respectively. Investigate its reason, RF is an ensemble learning method that improves generalization by combining multiple decision trees. It can handle high-dimensional data and feature interactions, reducing overfitting. Additionally, it possesses strong anti-overfitting capabilities by randomly selecting features and constructing decision trees through random sampling. Furthermore, it effectively deals with imbalanced datasets by focusing on minority class samples and provides interpretability and feature importance measurements, achieving outstanding performance in multi-class machine learning.
2. Max_depth is a key hyperparameter in the decision tree model. It can be seen that the selection of the hyperparameter max_depth has a certain impact on model training. This paper uses methods such as ten-fold cross-validation to try different max_depth values, and then select the model with the best performance on the data set. This is a common parameter adjustment method that can help us find optimal hyperparameter values. But for NBM, DBN, and BP Neural Network, the design of these models does not involve parameters such as “max_depth”, because their structure and training methods are different from other models that need to set depth or layer limit, so their training results Not affected by max_depth. It can be seen that when max_depth is 10, the effect of the machine learning model is usually better than when max_depth is 9 or 11. For the RF model, when max_depth is 10, the model accuracy of the left eye and right eye is 0.8775 and 0.8229 respectively, and the Macro F1 is 0.8019 and 0.8080 respectively. When max_depth is 9 and 11, the model accuracy of the left and right eyes is reduced by 1% and 1% on average, and Macro F1 is reduced by 4% and 3% on average. This suggests that at a maximum depth of 10, the model is better able to capture complex relationships in the training data, while a depth of 9 or 11 may be too simple or too complex, resulting in degraded performance. Choosing an appropriate max_depth is crucial for models such as random forests, which directly affects the complexity and generalization ability of the model.
3. Feature selection generally improves the performance of classification models, but there are exceptions. E.g, through the analysis of experimental results, it can be seen that NBM models are usually used to process high-dimensional data, and feature selection will reduce the number of features, thereby reducing the ability of NBM to capture complex relationships in the data. On high-dimensional data, selecting the right subset of features may become more difficult, reducing model performance.
4. Based on the results of the above comparative experiments, it can be observed that the prediction performance of the left-eye model in the proposed model was generally better than that of the right eye. We considered the order of surgical eye treatment and individual patient variability in physiological factors, such as axial length, corneal curvature, pupil size, occupation, and eye habits. Therefore, the dioptres corrected for each eyeball and the depth of the corneal cut will also vary, which will have some impact on the predictive effect of the type of surgery.

**Fig. 1:**
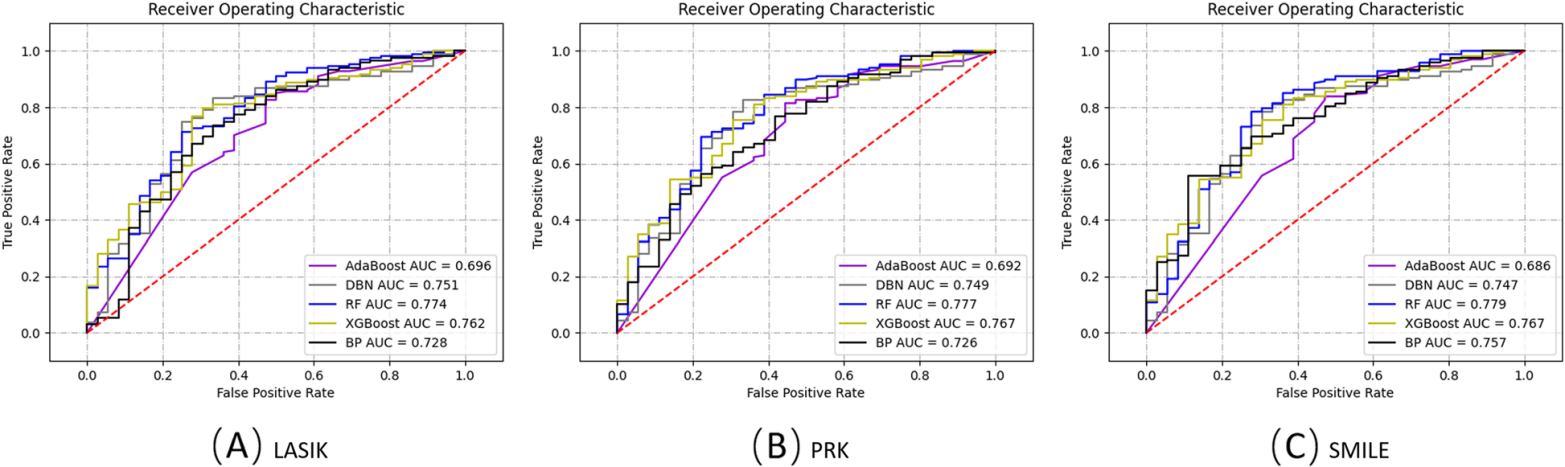
Comparison of the ROC curves of the five classification algorithms predicted for each surgical method in the data set. **A** LASIK. **B** PRK. **C** SMILE

Figure 1 is a comparison chart of the ROC curves of the five classification algorithms that predict each surgical method in the data set. The ROC curve shown in Fig. 1A shows the relationship between the specificity and sensitivity of the classifier when predicting whether a patient will undergo lasik surgery, and is a comprehensive representation of the diagnostic accuracy of the classifier. For different classifiers, the larger the area under the ROC curve (AUC), the better the diagnostic performance. As can be seen from the figure, the ROC area of the RF model is the largest, and the probability of being consistent with the doctor is the highest, proving that the model has good comprehensive performance.

#### Feature importance and visualization

**Fig. 2:**
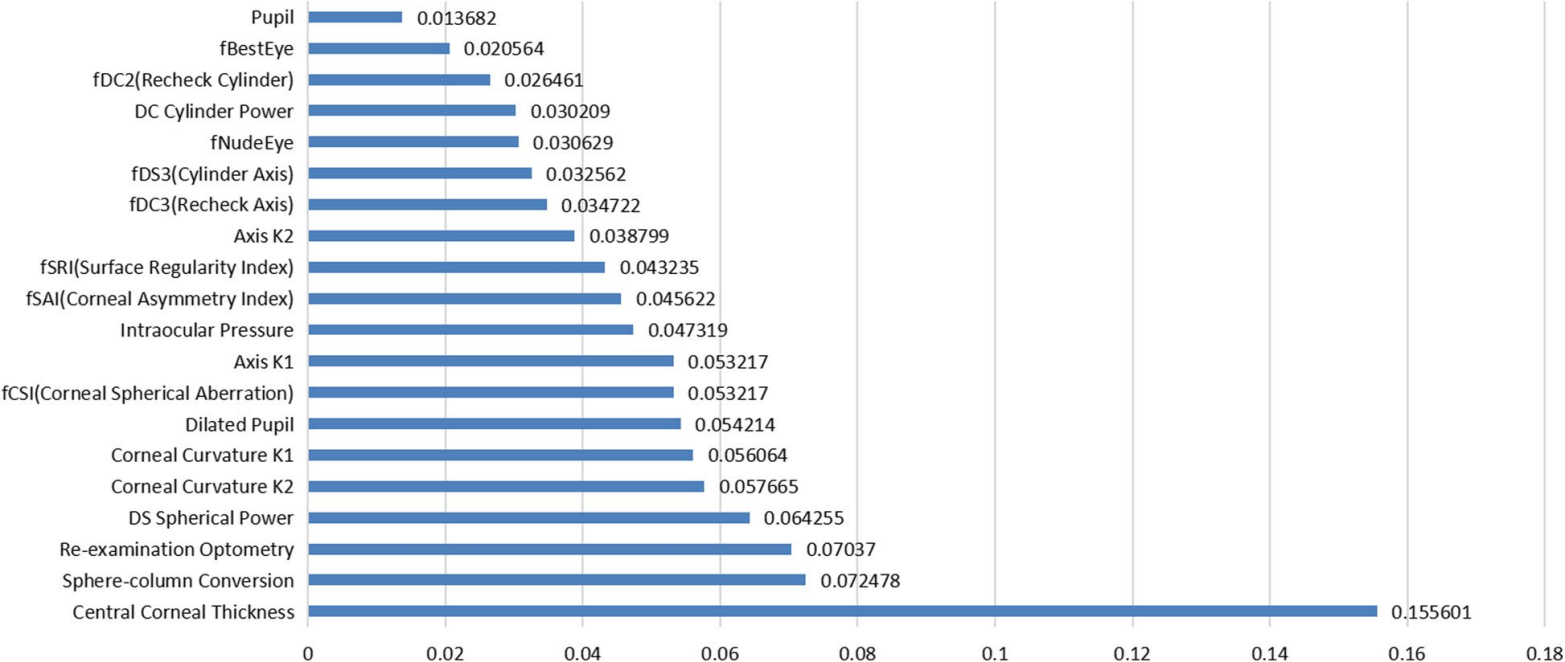
SHAP feature importance matrix map of random forest model

**Fig. 3:**
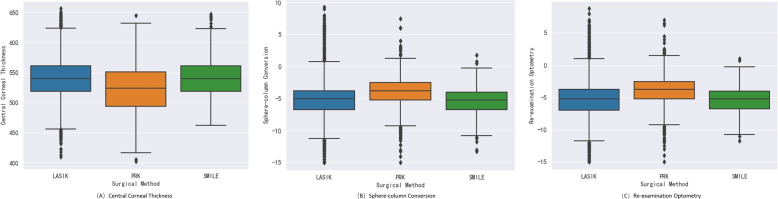
Box plot of data distribution of the top 3 variables of SHAP importance in each surgical method

In this section, we investigate the importance of each feature for classification. In Fig. 2 the SHAP feature importance matrix graph shows the features with high importance and quantifies the impact of multiple clinical features, such as Central Corneal Thickness (0.1556), Sphere-column Conversion (0.0725), Re-examination Optometry (0.0704), DS Spherical Power (0.0643), Corneal Curvature K2 (0.0577), and Corneal Curvature K1 (0.0561). All of which play important roles in the selection of corneal refractive surgery methods. It was verified that the explanation of feature importance using SHAP techniques is consistent with the clinical diagnosis of oculists. As shown in Fig. 3, these three box plots respectively show the center position and scatter range of the data distribution of the top-3 variables in SHAP importance in each surgical method. The figure clearly and intuitively shows that there are obvious differences in the distribution of the three variables in different surgical methods.

Analysis shows the data span is more than eighteen years, and surgical techniques have innovated rapidly over time. The central corneal thickness is an important indicator in corneal refractive surgery, which to a certain extent determines the range of degrees that can be corrected by surgery, and has an important impact on the feasibility, safety, correction effect, and postoperative stability of surgery. In addition, a patient’s best vision while wearing glasses provides a baseline for physicians in assessing surgical feasibility and predicting surgical outcomes, helping them assess the potential degree of visual improvement after surgery. To a certain extent, it may have an impact on the goal setting, surgical effect prediction, and surgical type selection of corneal refractive surgery.

The interpretation of the feature importance using the SHAP technique in this study was consistent with the ophthalmologist’s practical Experience. In addition, our study also find that the new clinical indicator of sphere-column conversion (sphere-column conversion = spherical equivalent refraction * astigmatism / 2) has a certain degree of impact on the predicted results of the surgical method, which will provide ophthalmologists with a new clinical tip.

**Fig. 4:**
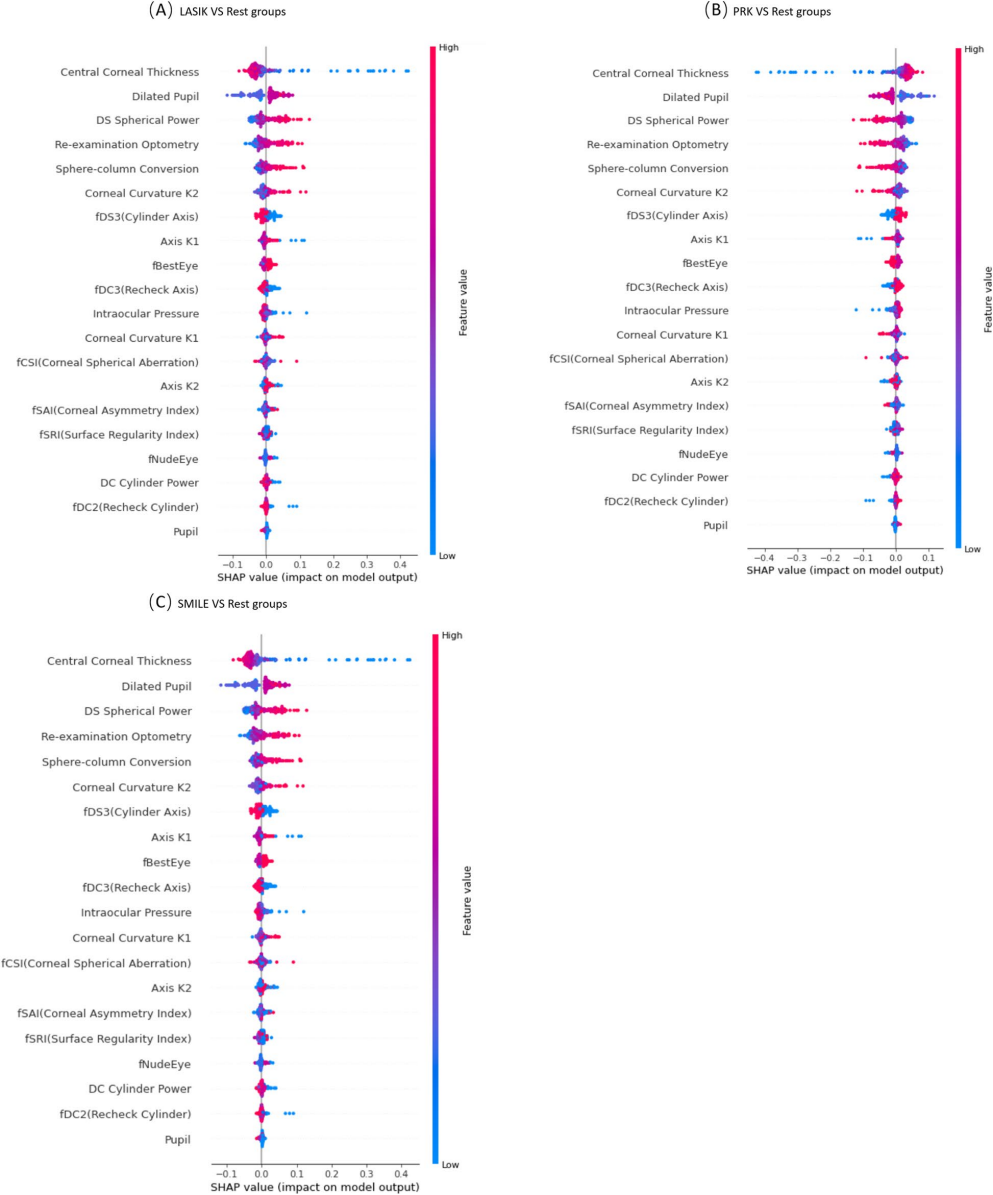
Random forest-based SHAP summary graph. **A** multiclass classification with LASIK versus rest groups. **B** multiclass classification with PRK versus rest groups. **C** multiclass classification with SMILE versus rest groups

Figure 4 shows the summary plot graphs of 3 random forest classifiers, each of which gives the global interpretability of why the corresponding surgical type was chosen. In each summary plot graph, the vertical axis sorts the features based on the sum of the SHAP values of all samples, while the horizontal axis represents the SHAP value, which is the distribution of the impact of the features on the model output. Each point represents a sample. From the origin to the right, the SHAP value is positive, indicating that the contribution of the feature to the prediction result is positive. The more lines to the right, the greater the contribution, and vice versa to the left.The thicker the line, the larger the sample size, and vice versa. The color from blue to red represents the representative value from small to large. As shown in Fig. 4, for all surgical types, Central Corneal Thickness, Dilated Pupil, DS Spherical Power, and Re-examination Optometry are the most important features.

**Fig. 5:**
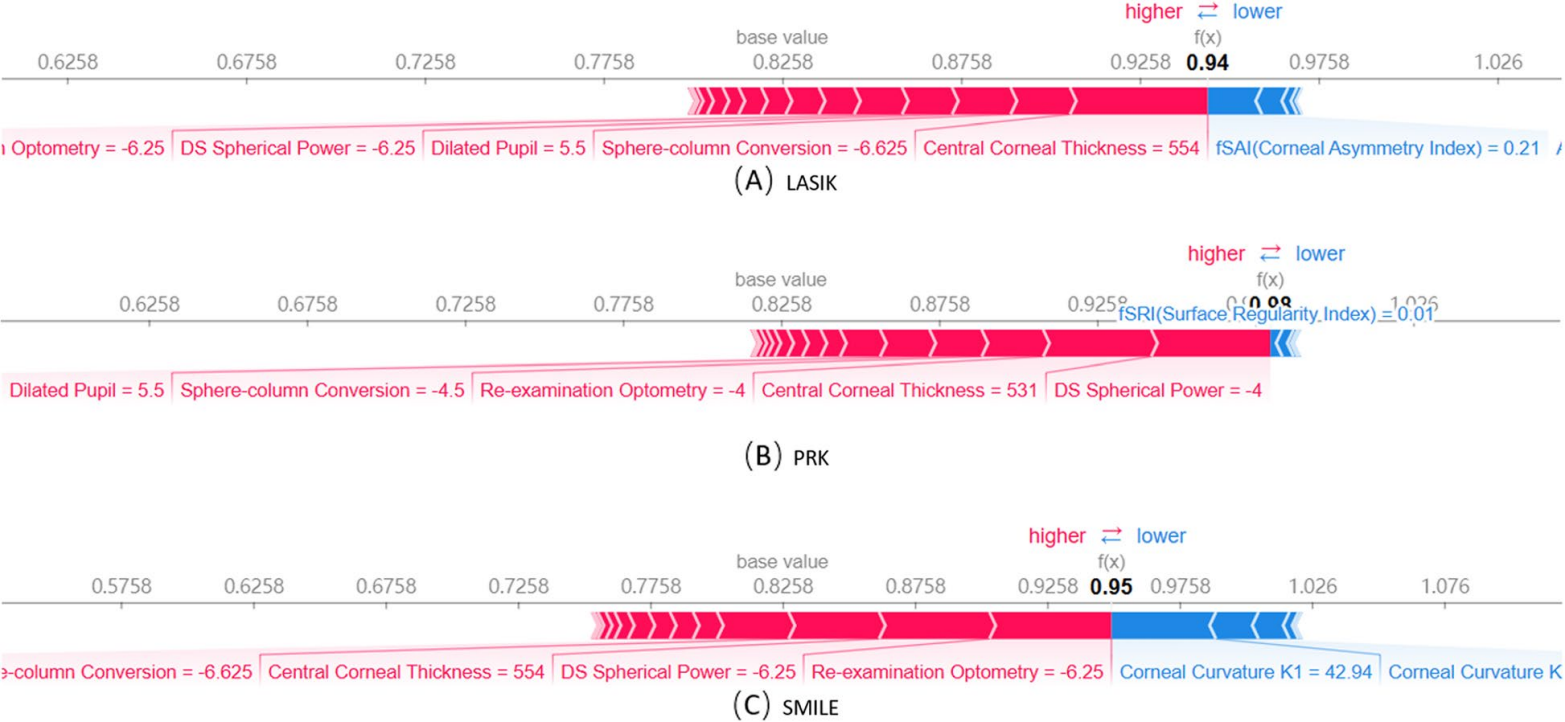
Case examples show the machine learning prediction results with local interpretation via force plots

Figure 5 illustrates a single-sample prediction explanation with a force plot. In the force plot, the SHAP value of each feature is visualized as a force that increases or decreases the prediction accuracy. The red force indicates a positive contribution to the predicted result, and the blue force indicates a negative contribution to the predicted result, while the quantity of contribution is expressed as a numerical value on the x-axis. E.g Fig. 4B indicates that the positive contribution of the feature of DS Spherical Power = -4 is the largest, followed by Central Corneal Thickness = 531, Re-examination Optometry = -4, Sphere-column Conversion = -4.5, and Dilated Pupil = 5.5, but the negative contribution of fSRI(Surface Regularity Index) = 0.01. This means that, under the comprehensive influence of all features, the probability that the model predicts that PRK should be used for this case is 98%. The fSRI in the patients with PRK surgery in the data set is mainly concentrated between [0.03, 0.20], but in this case, the Corneal Curvature K2 is 0.01, which has a negative impact on the classification model.

## Conclusion

In this paper, we proposed a machine learning-based prediction model for selecting corneal refractive surgery techniques. Firstly, we cleaned the data set, removed samples with missing key data, selected the top 12 features with feature importance greater than 1.4%, and solved the problem of data imbalance through SMOTE technology. Next, we selected six machine learning models and used 10-fold cross-validation and grid search to train the models and determine the optimal hyperparameters to improve model performance. The best method is to select the first 12 features with feature importance greater than 1.4% in the left eye, set max_depth to 10, and train the RF model through SMOTE, with an accuracy of 0.8775 and a Macro-F1 of 0.8019. Further, the SHAP technique is used to interpret feature importance consistent with the practical experience of ophthalmic surgeons. In our experiments, it was discovered and verified that the feature of “sphere-column conversion” had a certain degree of influence on the predicted results of the surgical method, which has new clinical application value.

In this research, 20 features including demographic characteristics, physical examination report, corneal biomechanical properties, ophthalmological measurements, and interview questionnaire were used, and 6 machine learning models were used for classifications. In further work, we will build multi-modal models to process various information including graphical data and text. At the same time, our analysis solely utilizes data from a single ophthalmic hospital, and the performance of the machine learning model may differ when applied to larger datasets with different patient features and institutions with different distributions. However, due to the limited amount of case data and the principle of confidentiality, we are unable to obtain external data. Ultimately, the developed model is able to provide confidence to doctors and patients by recommending surgery based on data when deciding on a surgical method.

The hospital’s ophthalmology department from which the data set in this study comes has high medical standards and high surgical quality, with an annual outpatient volume of more than 120,000 and an annual operation volume of more than 8,000. More than 98% of patients come to our center for review of uncorrected vision, corrected vision, subjective and objective refraction, and intraocular pressure, 1 day after surgery, 1 week after surgery, 1 month after surgery, 3 months after surgery, and 6 months after surgery. A slit lamp and other examinations were performed, and subjective refraction and dominant eye examination were performed in the 6th month after surgery. Because the time span of this study is very long, a small amount of postoperative examination data was missing, but the hospital conducted postoperative follow-up visits for all patients, and the results showed that the expected surgical results were achieved and there were no postoperative complications. This data set only records the surgical data of some doctors. These doctors have rich surgical experience and superb surgical skills, and there are no postoperative complications in this part. Data that could lead to postoperative complications were not recorded in this data set. Therefore, the failure to reflect surgical results such as postoperative complications, visual acuity, or refraction is a clear limitation of the study.

The data used in this study span a very long time span, more than 18 years. Combined with the very limited previous inspection methods, it is of great epochal significance to preserve well-preserved data and conduct research. Our next step is to obtain an external validation dataset, in order to prevent overfitting and better explore the effectiveness of the machine learning model across different institutions and surgeons.

## Data Availability

The datasets generated and/or analyzed during the current study are not publicly available but are available from the corresponding author upon reasonable request.
